# Cerebral small vessel disease and heart rate variability: A quest for nontraditional risk factors

**DOI:** 10.1111/jch.14350

**Published:** 2021-08-21

**Authors:** Aleksandra M. Pavlovic

**Affiliations:** ^1^ Faculty of Special Education and Rehabilitation University of Belgrade Belgrade Serbia

**Keywords:** heart rate variability, Mendelian randomization, risk factors, small vessel disease

The knowledge on cerebral small vessel disease (cSVD) risk factors, mechanisms, management, and prognosis has been expanded greatly in the last two decades. In this issue of the Journal of Clinical Hypertension, Tian and co‐workers investigated autonomic dysfunction as one of the nontraditional risk factors for cSVD occurrence and progression.^1^

We appreciate nowadays that neuroimaging markers of cSVD are a frequent finding in patients with common vascular risk factors, hypertension being the most prominent for its prevalence, impact, and potential for modifiability. Not only does cSVD cause a quarter of all ischemic strokes, typically leading to small subcortical infarctions and presenting with lacunar syndromes, but it is also the most common cause of vascular cognitive impairment and vascular dementia, in addition to being often detected in mixed dementias.^2^, ^3^ On the other hand, the increasing use of magnetic resonance imaging, which plays a key role in the diagnosis of SVD, leads to more frequent recognition of clinically silent lesions usually referred to as “covert SVD”.^4^ Nevertheless, covert cSVD are clinically relevant as they are increasing the risk of future stroke, cognitive impairment, dependency, and death.^2^, ^3^, ^4^ Great clinical and neuroimaging heterogeneity of the cSVD spectrum makes both the clinical and research approach to this topic even more challenging. For example, magnetic resonance imaging markers of cSVD are associated with more than doubled the risk for the transition from an autonomous to a dependent status after 3 years of follow‐up and are also associated with characteristic gait, motor, urinary, mood, and behavioral disturbances which although typical can be difficult to detect if mild.^2^, ^3^, ^4^ Nevertheless, at the point of the first evaluation, your patient may be sitting in front of you with no detectable clinical or cognitive manifestations but very worried while discussing with you abundant magnetic resonance imaging evidence of the disease found on their brain.

Sporadic cSVD is considered to be among the most common known neuropathological process of the brain but despite continuous and extensive research efforts, the exact sequence of pathological processes is not completely known, mechanisms linking vascular damage to small vessels with parenchymal damage of the brain are not fully elucidated, and clinical and neuroimaging correlation is often lacking.^2^, ^4^ Notably, cSVD is often diffuse but also dynamic, and there is still a need for a better understading of numerous aspects of this condition.^2^ The development of prevention and treatment strategies in cSVD is inevitably dependent on disease mechanisms, and there is an ongoing quest for neglected or underappreciated risk factors. The need for tracking of nontraditional risk factors is further underscored by the only moderate predictive value of traditional tools such as the ABCD2 score (age, blood pressure, clinical features, duration of symptoms, diabetes) in patients with a transient ischemic attack or minor stroke in general. Previous research provided evidence of autonomic dysfunction indicators in cSVD patients, such as 24‐h mean systolic BP (SBP), 24‐h mean diastolic BP (DBP), daytime mean SBP, nocturnal mean SBP, nocturnal mean DBP, but data on heart rate variability (HRV) have been controversial.^1^ The measurement of changes in HRV has been indicated as an important and novel tool for risk stratification and outcome prediction in patients with cardiovascular diseases, as it reflects the overall level of autonomic nervous system dysfunction.^6^ In study by Tian and colleagues, the genetic relationship between HRV and cSVD was explored by analyzing previous genome‐wide association studies data from the UK Biobank neuroimaging dataset and the MEGASTROKE Genome‐wise association study dataset, with the use of the Mendelian randomization (MR) method.^1^ The authors found that genetically predicted traits of HRV were associated with an increased risk of white matter hyperintensities but not SVD‐related stroke, reviving the discussion of different pathogenetic mechanisms in white matter hyperintensities and lacunar stroke.^1^, ^2^ Increased HRV was suggested to correlate with blunted nocturnal heart rate dipping, which may represent a state of sympathetic overdrive and occurs as a response to mental or physical stress, cardiac or noncardiac disease; it has been also associated with resistant hypertension and cardiovascular events.^1^, ^5^ Since it is suggested that autonomic imbalance plays a role in cSVD onset, a prediction model incorporating HRV could identify hypertensive patients at high risk of developing vascular events with higher sensitivity and specificity, indicating the need for increased exploitation of HRV in risk stratification tools of stroke, SVD in particular.^1^, ^5^

The causal relationship between HRV and white matter hyperintensities can be explained as an interplay of several mechanisms, comprising mechanical stress on the endothelium and its consecutive dysfunction due to an increased pulsatile flow by higher HRV, which occurrs in the particularly vulnerable vascular network in deep subcortical and periventricular brain regions lacking anastomoses, as well as failing autoregulation in the rigid vessels additionally contributing to hypoperfusion.^2^ It is an intriguing question why Tian and co‐workers’ study did not show the causal relationship between HRV and lacunar strokes,^1^ but an explanation of how SVD might produce different lesions in different locations of the brain and different segments of the vascular tree is hidden in details of the subtle yet powerful small vessel network. A very recent review of lacunar stroke mechanisms emphasized again the heterogeneous nature of this phenomenon and the various mechanisms involved, ranging from lipohyalinosis as the most common but also including atheromatous disease and cardioembolism.^6^

Mendelian randomization (MR) is increasingly being used to examine the causal relationship between exposure (e.g. risk factors) and outcome of interest (e.g. disease), as it is subject to less confounding than conventional observational data analyses when properly used. The method is based on Mendel's law on the random assortment of genetic variants, so it uses genetic variants as instrumental variables in an approach that mimics the main principle of randomization applied in clinical trials. In the field of cSVD, the MR has been most recently used in exploring the relationship between total homocysteine levels and cSVD imaging burden,^7^ diabetes mellitus and glycemic traits, and small vessel stroke^8^, as well as serum matrix metalloproteinase‐8 levels and ischemic stroke subtypes.^9^ Interesting conclusions were derived from a recent analysis by Harshfield and colleagues, who assessed the causal effect of 12 lifestyle factors on the risk of stroke using the MR method.^10^ In terms of small vessel stroke, there was genetic evidence that reduced education and increased smoking and obesity increase the risk of small vessel stroke, suggesting that lifestyle modifications addressing these risk factors will reduce stroke risk.^10^ However, the MR method does not serve as a substitute for a well‐designed randomized clinical trial.

The global burden of cSVD remains important as a powerful predictor of long‐term cognitive decline and functional disability. The European Stroke Organisation recently presented guidelines on covert cSVD,^4^ while the management of different aspects of overt SVD has recently been reviewed in several publications.^3^, ^6^, ^11^ Addressing cardiovascular risk factors is currently the most effective approach to managing both overt and covert cSVD. With this goal in mind, there is a potential for genetic stratification of high‐risk patients and genetically assisted selection of drug targets in prevention trials.^12^ These findings might be opening a new door to more advanced personalized interventions in our patients at risk of vascular damage to the brain affecting the small vessels network.^12^ When the quest for nontraditional risk factors for cSVD will end and how we will translate this information into real‐life clinical practice and individual patient decisions remains to be seen.

## FUNDING

None

## CONFLICT OF INTEREST

The author has no conflict of interest to declare

## AUTHOR CONTRIBUTION

I, Aleksandra Pavlovic, state that I am the only author of this manuscript, which has been solely written by me.

Aleksandra M. Pavlovic

Belgrade, Serbia July 20th 2021
